# A Deep Learning-Based Method for Stress Measurement Using Longitudinal Critically Refracted Waves

**DOI:** 10.3390/s26072283

**Published:** 2026-04-07

**Authors:** Yong Gan, Jingkun Ma, Binpeng Zhang, Yang Zheng, Xuedong Wang, Yuhong Zhu, Yibo Wang, Dachun Ji

**Affiliations:** 1 School of Mechanical and Electrical Engineering, Guilin University of Electronic Technology, Guilin 541004, China; 2 Key Laboratory of Nondestructive Testing and Evaluation, State Administration for Market Regulation, China Special Equipment Inspection and Research Institute, Beijing 100029, China; 3 Qingdao Haier Washing Machine Co., Ltd., Qingdao 266100, China; 4 School of Information and Communication Engineering, North University of China, Taiyuan 030051, China

**Keywords:** stress measurement, deep learning, longitudinal critically refracted waves, ultrasonic, convolutional neural network

## Abstract

Accurate stress measurement is essential to evaluating structural integrity and plays a pivotal role in the health monitoring and predicting the service life of steel infrastructures. This study proposes a deep learning approach for stress prediction based on longitudinal critically refracted (LCR) ultrasonic waves. The model integrates gated recurrent units (GRU), attention mechanisms, and one-dimensional convolutional neural networks (1D-CNN), enabling direct stress prediction from raw ultrasonic signals without the need for manual feature extraction or explicit physical modeling. To validate the approach, LCR signals were acquired using a custom-built piezoelectric ultrasonic system from 20# steel specimens subjected to uniaxial stresses ranging from 0 to 200 MPa. A dataset comprising 4200 samples was augmented to enhance training efficiency. The proposed model achieved a mean absolute error of 1.94 MPa. Generalization tests demonstrated high accuracy across diverse stress levels, with average errors below 3 MPa, highlighting the model’s robustness. This research presents an accurate, intelligent, and calibration-free ultrasonic method for stress evaluation, providing practical support for stress evaluation in steel structures under actual operating conditions.

## 1. Introduction

Steel structures are extensively employed in aerospace, heavy machinery, oil pipelines, and automotive manufacturing owing to their high strength and seismic resilience [1,2]. These systems often operate under continuous loading, during which fatigue, thermal cycling, and external impacts induce stress accumulation in critical components. Such stress accelerates the degradation of material properties and heightens the risk of structural failure [3]. Accurate stress assessment is therefore vital for maintaining structural integrity.

Common nondestructive testing (NDT) methods for stress evaluation include strain gauge techniques [4], X-ray diffraction [5], magnetic methods [6], and ultrasonic techniques [7]. Strain gauges are simple and cost-effective but measure only surface stress and are susceptible to environmental interference, limiting their long-term reliability. X-ray diffraction provides high accuracy but requires complex, expensive instrumentation and strict operational conditions. Magnetic methods apply only to ferromagnetic materials. Advancements in acoustic and ultrasonic technologies have significantly driven progress across various complex engineering domains [8,9,10]. By contrast, ultrasonic techniques enable nondestructive and accurate measurement of both surface and internal stress. Its lightweight, low-cost instrumentation and broad material applicability make them particularly promising approach for stress evaluation [11].

Longitudinal critically refracted (LCR) waves exhibit well-defined propagation paths and high sensitivity to stress variations, making them a preferred wave mode for stress analysis. These advantages have attracted significant interest from researchers. Li et al. [12] proposed a stress evaluation method for steel components based on a cross-correlation algorithm applied to LCR waves. This method surpasses traditional peak-based methods in accuracy and eliminates the need for signal filtering, presenting an effective solution for practical stress measurement. Lu et al. [13] developed a stress characterization model based on LCR waves and achieved the measurement of the depth distribution of residual stress within aluminum alloys. Zhao et al. [14] employed LCR waves to measure stress in steel structures, demonstrating the feasibility of rapid, non-destructive detection of initial internal stress. Their study also revealed a linear correlation between stress magnitude and time of flight. Li et al. [15] proposed a one-transmit, two-receive transducer array technique incorporating temperature–stress coupling for absolute stress measurement in steel components. The method achieved measurement errors within 10 MPa. Ma et al. [16] investigated complex residual stress distributions in metal plates using LCR waves based on acoustoelastic effect, demonstrating that LCR waves are feasible for stress measurement. Liu et al. [17] developed an ultrasonic absolute stress measurement system integrating an optimized wavelet transform with a cross-correlation algorithm. The system achieved an accuracy error less than 23 MPa and a stability error of 4.94 MPa in stress measurement of steel components.

The above-mentioned research has demonstrated the feasibility of nondestructive stress measurement using LCR waves. However, existing methods often depend on physical models developed under idealized conditions and require manual feature extraction along with extensive data preprocessing. These limitations reduce detection efficiency and hinder the deployment of rapid, real-time stress measurement in engineering environments.

Deep learning (DL) algorithms provide effective solutions for accurate stress measurement using ultrasonic by overcoming key limitations of traditional ultrasonic methods, including limited adaptability to complex conditions, reliance on manual intervention, and low detection efficiency. Pradhan et al. [18] proposed a model that integrates machine learning with ultrasonic to measure surface residual stress during the rolling process of lightweight alloys. The model achieved high determination coefficients (R^2^) of 0.973 for compressive stress and 0.926 for tensile stress, with relatively low root mean square errors. Lim et al. [19] integrated Lamb waves with convolutional neural networks to directly predict stress from ultrasonic signals, eliminating the need for acoustic feature extraction in traditional methods. The approach exhibited strong robustness under both static and dynamic loading conditions up to 5 Hz, validating the effectiveness of the model. Park et al. [20] applied machine learning to ultrasonic amplitude-scan signals propagated through the aluminum alloy to nondestructively estimate a full-range stress–strain curve, establishing a quantitative relationship between ultrasonic characteristics and mechanical performance. Deng et al. [21] proposed an absolute stress identification method that integrates a one-dimensional convolutional neural network (1D-CNN) with ultrasonic shear waves. The method achieved an average relative error of 3.83% and demonstrated strong adaptability to steel components of varying thicknesses.

These studies demonstrate that DL-based stress measurement methods can directly map ultrasonic signals to stress values, reducing dependence on manual acoustic feature extraction and eliminating the need for physical modeling in ultrasonic stress measurement. However, existing DL methods for stress measurement are constrained by limited accuracy and poor generalizability. Moreover, few studies have focused on LCR waves, and the advantages of their clear propagation paths and high sensitivity to stress variations have not been fully exploited.

To address this gap, this study progressed as follows. A nondestructive testing experimental platform utilizing LCR waves was established to acquire ultrasonic signals under varying stress conditions. An end-to-end DL model was then developed by integrating 1D-CNN, gated recurrent units (GRU), and an attention mechanism to directly map ultrasonic signals to stress values. Furthermore, data augmentation techniques were employed to expand both the size and diversity of the dataset. Based on this framework, a high-precision stress measurement model for LCR waves was developed. Generalization experiments confirmed the model’s strong robustness and stability under varying conditions.

## 2. Theoretical and Experimental Studies

### 2.1. Principle of LCR Waves

When ultrasonic waves encounter the interface between two media, both reflection and refraction occur. These processes are determined by the angle of incidence. At the critical angle, a portion of the refracted wave propagates along the interface, traveling near the boundary within the second medium. This phenomenon generates an LCR wave [22].

The phenomenon of critical refraction is described by Snell’s law, which can be expressed as:

(1) $$\frac{\sin\theta_{1}}{v_{1}}=\frac{\sin\theta_{2}}{v_{2}}$$

The critical refraction condition is given by *θ*_2_ = 90°.

(2) $$\theta_{\mathrm{crit}}=\arcsin\left(\frac{v_{1}}{v_{2}}\right)$$

**Figure 1:**
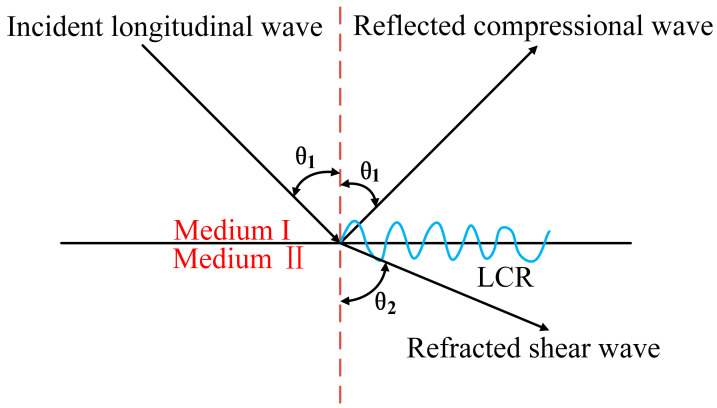
Schematic diagram of longitudinal critically refracted (LCR) wave propagation principle.

Here, *θ*_1_ denotes both the angle of incidence and the first critical refraction angle, *θ*_2_ represents the refraction angle, and *v*_1_ and *v*_2_ correspond to the acoustic velocities in the first and second media, respectively. The propagation mechanism is illustrated in Figure 1.

**Figure 2:**
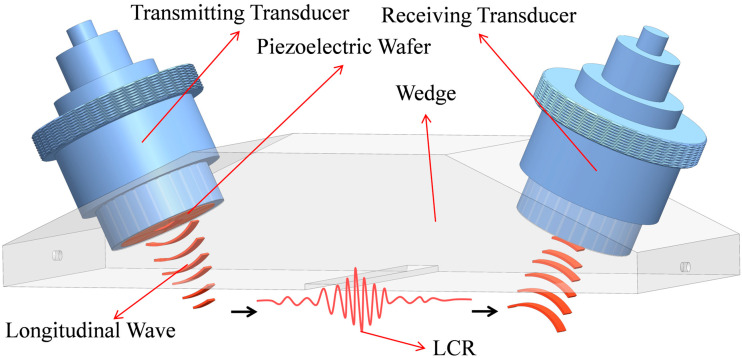
Schematic diagram of the LCR excitation structure.

The velocity of LCR waves is highly sensitive to stress variations, making them well suited for stress measurement. In the experimental setup, LCR waves are typically generated using a wedge, with its angle designed according to the first critical angle to ensure that the transducer can excite longitudinal critically refracted waves. The three-dimensional schematic diagram of the transducer structure is shown in Figure 2.

### 2.2. Experiments

#### 2.2.1. Experimental Platform

**Figure 3:**
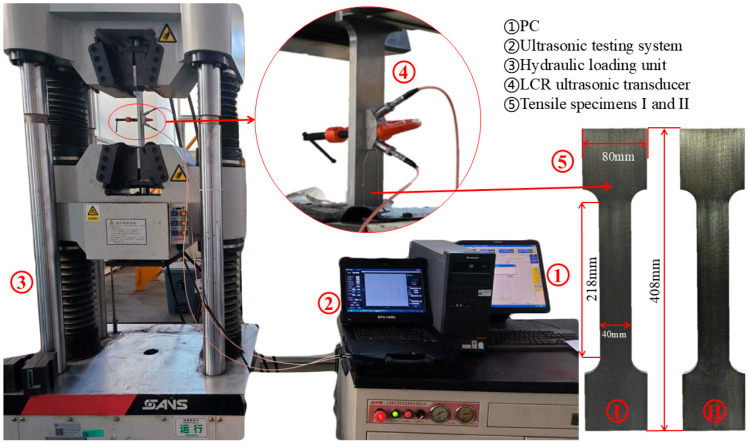
Overall experimental system.

A piezoelectric ultrasonic experimental platform was developed for stress measurement, and the experimental platform configuration was illustrated in Figure 3. The system employed LCR transducers for both excitation and reception of ultrasonic signals. The test specimens were standard tensile samples of 20# steel, each with a thickness of 20 mm and a gauge section of 200 mm × 40 mm. To ensure the consistency of the initial material state and to minimize the interference of microstructural variability, all specimens were machined from the same production batch of 20# steel. Furthermore, the loading axis of each specimen was aligned parallel to the rolling direction of the raw sheet. This strict preparation protocol was implemented to maintain a consistent crystallographic texture across the dataset, thereby isolating the stress state as the primary variable for the deep learning model. At the same time, to ensure both applicability and rigor, two specimens of identical geometry and dimensions but differing service conditions were selected: Specimen I, a brand-new sample, and Specimen II, a previously used one.

**Figure 4:**
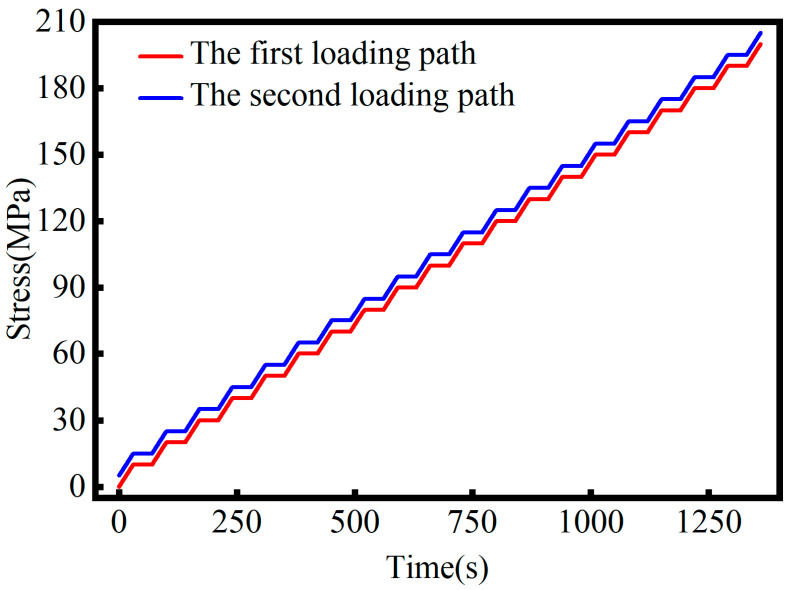
Stress loading process for the two loading strategies.

During the experiment, uniaxial tensile loads were applied to standard metal specimens using a Personal Computer (PC)-controlled SHT4106 hydraulic loading unit (Shenzhen SANS Testing Machine Co., Ltd., Shenzhen, China), enabling precise control of the stress gradient. The applied stress ranged from 0 to 200 MPa, with each 10 MPa increment corresponding to approximately 80 kN of tensile force. To broaden stress coverage, two loading paths were designed. The first path (illustrated in Figure 4) involved stepwise loading from 0 to 200 MPa in 10 MPa increments, starting at 0 N and increasing by 80 kN per step, generating 20 stress levels. The second path (illustrated in Figure 4) ranged from 5 to 195 MPa with identical increments, beginning at 40 kN and also yielding 20 stress levels. Each loading path was applied to specimens in two different service states, resulting in four loading tests in total. Collectively, these protocols ensured uniform stress distribution and produced a robust dataset for model training.

**Figure 5:**
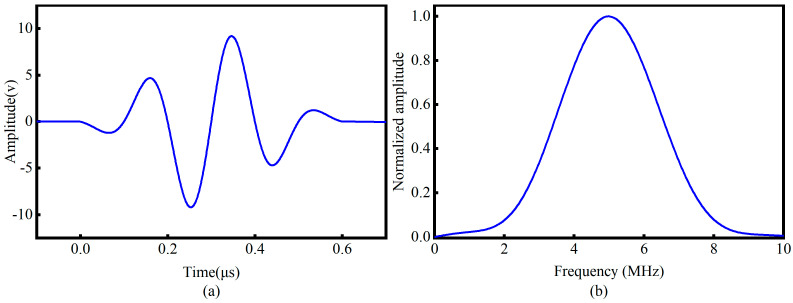
(**a**) Time domain waveform of the excitation signal. (**b**) Frequency domain spectrum of the excitation signal.

Moreover, as shown in Figure 3, at each stress level a pair of piezoelectric transducers, configured in a transmit-receive arrangement, was used to generate and capture LCR waves. The excitation signal (shown in Figure 5) was a three-cycle 5 MHz sine wave, with the pulse width adjusted to optimize the transducer’s frequency response. The excitation voltage was set to 10 V, and the receive-channel gain fixed at 65 dB. To ensure that the variations in the LCR waves are directly induced by the applied stress, the transmit-receive axis of the transducers was aligned parallel to the loading axis of the specimen, making the LCR wave propagation direction strictly parallel to the uniaxial tensile stress. Transducers were mounted on the specimen surface using custom fixtures, with honey served as the coupling medium to enhance acoustic transmission intensity and minimize variability due to coupling instability.

In this study, the transducers were rigidly mounted using custom fixtures throughout the loading process. This configuration was strategically chosen to prioritize the acquisition of a high-fidelity ‘stress-signal’ dataset, ensuring that signal variations were exclusively driven by stress changes rather than operational inconsistencies. Manually re-coupling the transducer at each load step was excluded to eliminate random errors during the ground-truth data collection phase and to mitigate safety risks associated with manual intervention during active hydraulic loading.

All experiments were conducted under ambient laboratory temperature conditions. Furthermore, the coordination between the loading paths and the instantaneous data acquisition scheme ensured that each experimental cycle was completed within a minimized time window. Consequently, ambient temperature variations during this brief period were negligible and thus ignored.

**Figure 6:**
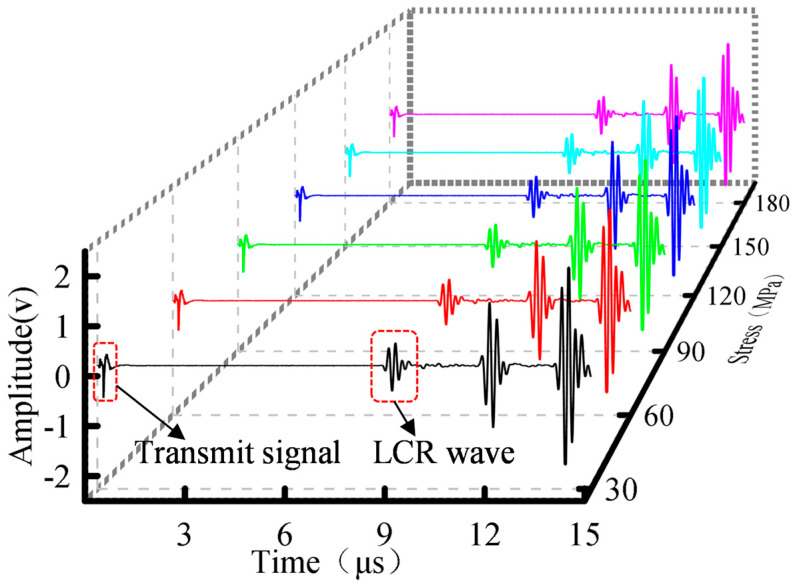
Time domain waveforms of received signals under different stress levels.

Signals were sampled at 1 GHz with 15 k points per acquisition, capturing both the excitation response and multiple echoes. To preserve signal integrity, no digital filtering or averaging was applied during acquisition. As shown in Figure 6, this excitation-reception scheme produced clear, energy-concentrated ultrasonic echoes. Specifically, the first arriving wave packet is the LCR wave, which propagates along the surface at the maximum acoustic velocity. The two subsequent wave packages correspond to the longitudinal wave and the transverse wave reflecting from the specimen bottom, respectively. This provides a robust foundation for subsequent analysis and feature extraction.

At each stress level, five ultrasonic signals were repeatedly acquired, and the corresponding tensile stress values were recorded to establish a labeled dataset. In total, 420 signals were collected, providing high-quality datasets for training and evaluating the DL model.

#### 2.2.2. Data Preprocessing and Training Set Construction

Adequate sample size is essential for the reliable performance of the DL model [23]. Insufficient data commonly leads to overfitting, thereby limiting a model’s ability to generalize and make accurate prediction [24]. Expanding the dataset increases training diversity and improves the capacity to capture feature variations across different environments. To address the limited size of the training set, data augmentation methods were applied to expand the initial dataset to 4200 samples [25]. The augmentation process introduced random noise, temporal scaling, and amplitude scaling. These adjustments preserved the essential signal structure while increasing data diversity. This strategy enhanced model robustness to varying conditions and noise, thereby improving predictive accuracy on novel datasets.

**Figure 7:**
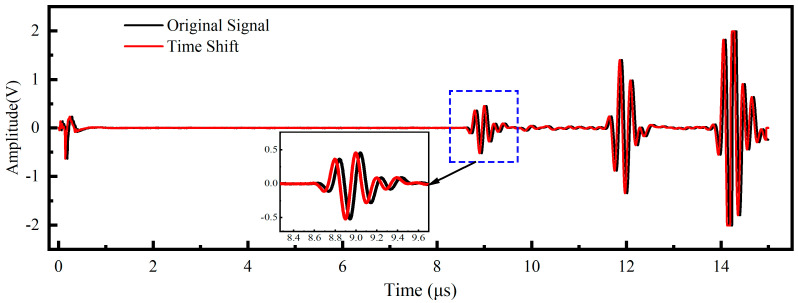
Data augmentation via random time shifting (range: ±50 ns).

**Figure 8:**
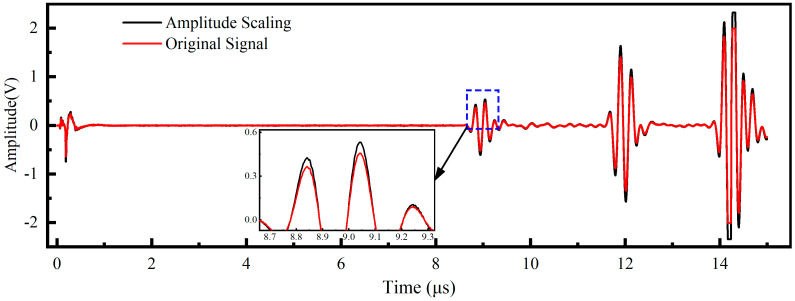
Simulation of coupling variability using random amplitude scaling (factor: 0.5–1.5).

**Figure 9:**
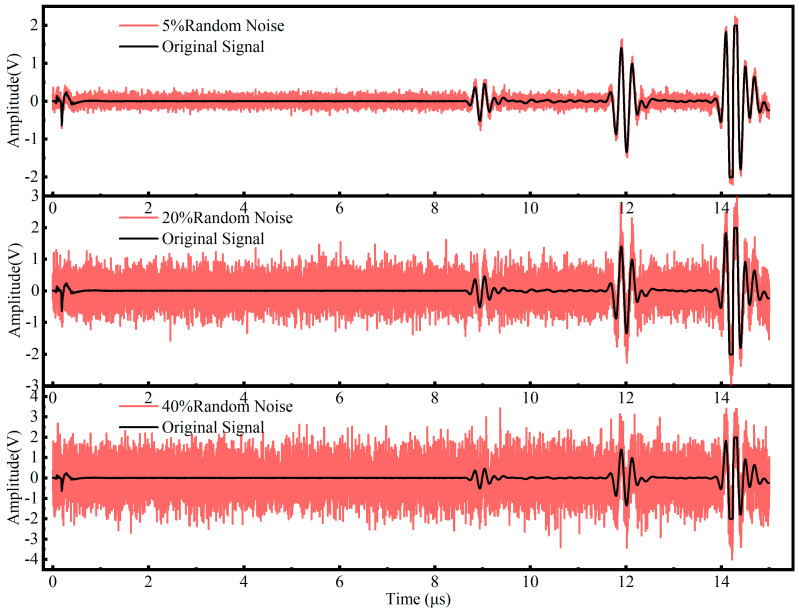
Gaussian white noise injection at varying intensity levels (std: 5–40%).

Specifically, first, temporal triggering jitter was simulated by applying a random time shift to the raw signals [26]. As illustrated in Figure 7, the waveforms were translated along the time axis within a range of −50 to +50 sampling points, which corresponds to a temporal window of ±50 ns. This process ensures the model remains robust against minor synchronization errors. Second, variations in coupling layer thickness and fixture pressing force were modeled using random amplitude scaling [27]. Figure 8 depicts this process where the signal magnitude was multiplied by a stochastic factor uniformly distributed between 0.5 and 1.5. This mathematical operation effectively mimics the energy fluctuations caused by inconsistent sensor contact. Third, environmental interference and electronic instability were introduced by injecting Gaussian white noise into the original signals [23]. Figure 9 demonstrates the effect of noise injection at varying intensity levels. The noise standard deviation was randomized to fall between 5% and 40% of the maximum signal amplitude. This procedure forces the neural network to extract intrinsic stress features despite low signal-to-noise ratio conditions.

Each ultrasonic signal, whether original or augmented, was represented as a one-dimensional time series of 15 k data points. To mitigate scale inconsistencies arising from variations in amplitude and energy, all signals were standardized using the StandardScaler algorithm, which normalizes each signal to zero mean and unit variance. This preprocessing step enhanced feature consistency, accelerated convergence, and improved training stability. The dataset was then partitioned into training, validation, and test sets in an 8:1:1 ratio. The training set was used for model learning, the validation set for performance monitoring and hyperparameter tuning, and the test set for assessing generalization. To reduce potential bias, stress levels were balanced across all subsets.

The expanded dataset, constructed through the aforementioned data augmentation methods, was converted to tensor format and integrated into the PyTorch (2.9.0) framework for model training. During data loading, random shuffling was applied to further improve model robustness. By explicitly exposing the 1D-CNN-GRU-Attention network to these simulated deviations during the training phase, the model is trained to maintain stable feature extraction and high prediction accuracy, even when encountering similar signal distortions in practical environments. Additionally, the large volume of the augmented dataset effectively enhances the convergence capability of the model.

## 3. Model Development

### 3.1. Design of the Neural Network Model

**Figure 10:**
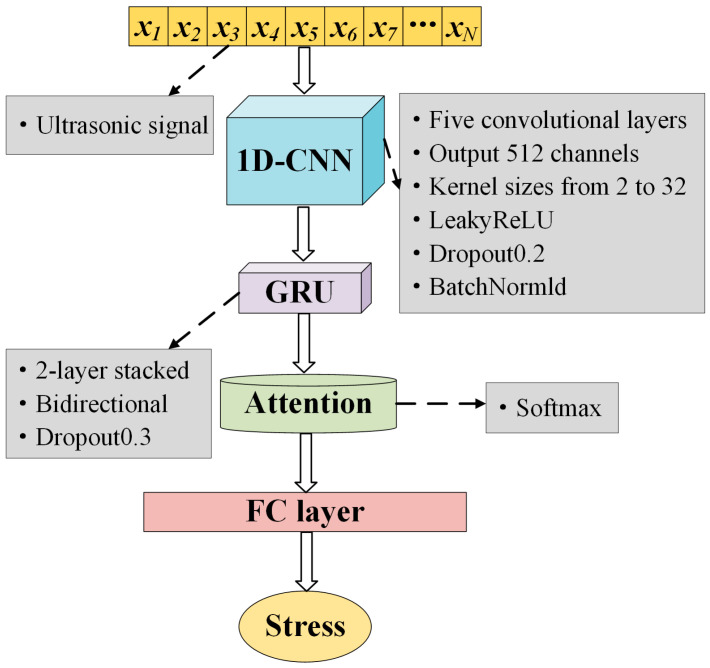
Architecture and corresponding parameters of the CNN-GRU-Attention neural network model.

Ultrasonic signals for stress measurement are high-resolution and non-stationary, exhibiting pronounced local fluctuations. Stress-related information often appears as subtle and localized perturbations. Therefore, effective feature extraction plays a crucial role in improving the accuracy and robustness of the model. CNNs offer advantages in parameter sharing and local feature extraction, which helps mitigate overfitting caused by high-dimensional input data. And CNNs avoid time step unfolding, thereby improving training efficiency and enhancing the ability to capture local variations [28]. Consequently, CNNs were adopted as the core architecture to extract local features closely associated with stress variations. Additionally, recent studies have shown that hybrid deep learning architectures combining CNNs with recurrent networks such as LSTM or Bidirectional Long Short-Term Memory (BiLSTM) yield superior performance in capturing the complex spatiotemporal dynamics of ultrasonic signals for nondestructive evaluation [29,30,31]. Inspired by the proven benefits of these hybrid models, a deep hybrid network was constructed by integrating CNN, GRU, and an attention mechanism, as shown in Figure 10. The architecture of the model is specifically designed to correspond directly to the physical characteristics of LCR waves under stress.

The 1D-CNN is utilized for Local Feature Extraction. Stress induces micro-structural changes in the material, leading to subtle distortions in the LCR waveform envelope and frequency content. The 1D-CNN layers apply sliding convolutional filters to capture these local morphological features, effectively extracting the non-linear acoustoelastic response embedded in the waveform shape, as demonstrated in similar 1D signal classification tasks [32].

The GRU is employed for Temporal Evolution Modeling. The primary manifestation of stress is the alteration of acoustic velocity, which results in a temporal shift in the signal sequence. The GRU network is specialized for time series data as it captures the temporal dependencies and dynamic evolution of the signal [33]. This effectively models the precise time shift information that correlates with stress magnitude.

The attention mechanism is applied for Signal Saliency. In practical detection, the LCR wave constitutes only a specific segment of the acquired signal, often surrounded by scattering noise and other mode reflections. The attention mechanism mimics the human expert’s focus by assigning adaptive weights to the time steps. It highlights the stress-sensitive LCR wave packets, which are high-information regions and suppresses the irrelevant background noise, thereby improving the signal-to-noise ratio and prediction accuracy.

#### 3.1.1. 1D-CNN

**Figure 11:**
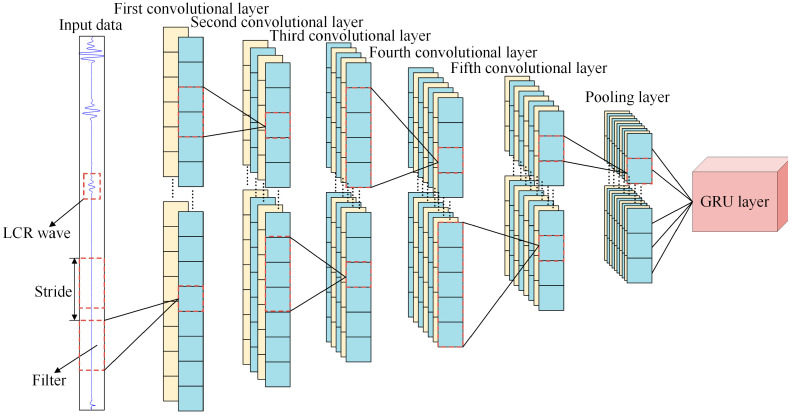
Internal architecture of the 1D-CNN.

1D-CNN is a deep model tailored for time series signals, consisting of convolutional layers, activation functions, normalization, and downsampling modules. By applying sliding convolutions along the temporal axis, the 1D-CNN automatically extracts local features such as amplitude discontinuities and periodic fluctuations. This architecture offers strong translation invariance and local perceptual ability, making it well suited for stress-related signal analysis. The specific 1D-CNN architecture adopted in this study is illustrated in Figure 11.

Within the convolutional layers, the 1D-CNN applies sliding kernels to perform localized weighted operations on input signals, thereby extracting temporal features and producing one-dimensional feature maps. Weight sharing along the temporal axis allows identical patterns to be detected at different positions, improving computational efficiency and enhancing local feature extraction. Let the input to the *l*-th layer be$\;x^{l-1}$, the processed output from the (*l* − 1)-th layer. The output of the *l*-th layer, denoted as $x^{l}$, represents the corresponding feature map. If the *l*-th layer contains *M* input units, a convolution kernel size of *k*, and a bias term $b_{j}^{l}$, the convolutional operation is expressed as shown in Equation (3).

(3) $$x_{j}^{l}=f\left(\sum_{i=1}^{M}x_{i}^{l-1}*k_{ij}^{l}+b_{j}^{l}\right)$$

Here, $x_{i}^{l-1}$ denotes the *i*-th input unit of the (*l* − 1)-th layer; $k_{ij}^{l}$ represents the weight matrix between the *i*-th input channel and the *j*-th convolution kernel in the *l*-th layer; *M* is the number of input channels in $x^{l-1}$; *f*( ) denotes the activation function; and * represents the one-dimensional convolution operation. Through weighted summation, the convolution kernel $k^{l}$ extracts local features from the input signal within its receptive field. The bias term $b_{j}^{l}$ is added to the convolution result to further refine the output. Finally, the result is passed through the non-linear activation function *f*, yielding the output $x_{i}^{l}$ of the convolutional layer.

#### 3.1.2. GRU

**Figure 12:**
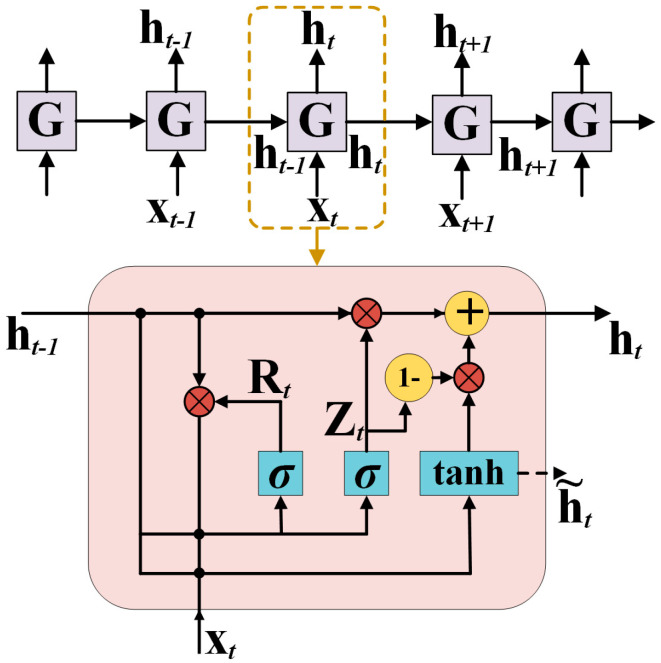
The basic architecture of the GRU network.

Traditional Recurrent Neural Networks (RNNs) suffer from vanishing gradients and limited memory, restricting their ability to capture long-term dependencies in complex time series data. To address these limitations, long short-term memory (LSTM) networks were developed, incorporating gating mechanisms that enhance information retention [34]. However, the intricate architecture of LSTMs introduces numerous parameters, leading to time-consuming training. To improve efficiency, Dey et al. introduced the GRU in 2017 [35], which simplifies the structure while maintaining strong temporal modeling capabilities. The GRU architecture is illustrated in Figure 12.

To capture temporal features and global dynamic dependencies in ultrasonic signals, a GRU module was incorporated after the convolutional layers that extract local features. For an input sequence {*x*_1_, *x*_2_, …, *x_t_*}, where each *x_t_* denotes the CNN-derived feature at time step *t*, the GRU updates its hidden state through a series of gating operations. The computations at each step are defined by Equations (4)–(7):

(4) $$Z_{t}=\sigma (W_{z}x_{t}+U_{z}h_{t-1}+b_{z})$$

(5) $$R_{t}=\sigma (W_{r}x_{t}+U_{r}h_{t-1}+b_{r})$$

(6) $${\tilde{h}}_{t}=\tanh(W_{h}x_{t}+U_{h}(R_{t}⊙h_{t-1})+b_{h})$$

(7) $$h_{t}=(1-Z_{t})⊙h_{t-1}+Z_{t}⊙{\tilde{h}}_{t}$$

In these equations, *Z_t_*, *R_t_*,$\;{\tilde{h}}_{t}$, and *h_t_* represent the update gate, reset gate, candidate hidden state, and final hidden state, respectively. *W* and *b* represent the weight matrices and bias vectors. The function tanh is the hyperbolic tangent activation, *σ* is the sigmoid function, and ⊙ denotes the Hadamard (element-wise) product. The update gate *Z_t_* regulates the balance between retaining information from the previous *h_t_*_−1_ and incorporating the candidate state ${\tilde{h}}_{t}$. The reset gate *R_t_* controls the extent to which the previous hidden state *h_t_*_−1_ is forgotten when computing ${\tilde{h}}_{t}$. The candidate hidden state ${\tilde{h}}_{t}$ is generated from the current input *x_t_* and the reset-modulated previous state via the tanh activation. Finally, the hidden state *h_t_* is obtained as a weighted combination of *h_t_*_−1_ and ${\tilde{h}}_{t}$ with weights governed by *Z_t_*. All parameters are optimized through backpropagation.

#### 3.1.3. Attention Mechanism

An attention mechanism was incorporated into the model to heighten its sensitivity to salient temporal features, thereby improving the accuracy of stress prediction. Ultrasonic signals typically comprise temporal segments that are informative for stress estimation, while others may be irrelevant or dominated by noise. The attention mechanism automatically assigns relevance weights to each temporal step, amplifying salient features and suppressing redundant or noisy components. Conceptually, it performs a weighted aggregation driven by correlation scores between sequence elements and a query vector. Given an input sequence {*x*_1_, *x*_2_, *…*, *x_t_*} and a query vector *q*, the relevance of each element *x_t_* is evaluated using a scoring function. A widely used formulation is the additive attention mechanism, expressed as follows:

(8) $$score(x_{t},q)=v^{T}\cdot \tanh(W_{1}x_{t}+W_{2}q)$$

In Equation (8), *W*_1_ and *W*_2_ represent learnable weight matrices, *v* represents a trainable context vector, and tanh is the hyperbolic tangent activation function. Next, the Softmax function is applied to normalize the relevance scores, producing attention weights for each temporal step. This normalization constrains the weights to form a probability distribution over the sequence, enabling the model to adaptively emphasize the most informative temporal components.

(9) $$\alpha_{t}=\frac{\exp(\mathrm{score}(x_{t},q))}{\sum_{t=1}^{T}\exp(\mathrm{score}(x_{t},q))}$$

Finally, a weighted summation of the input sequence is performed using the computed attention weights, producing an attention-enhanced representation that emphasizes temporally salient features while suppressing irrelevant information.


(10) $$c=\sum_{t=1}^{T}\alpha_{t}\cdot x_{t}$$

**Figure 13:**
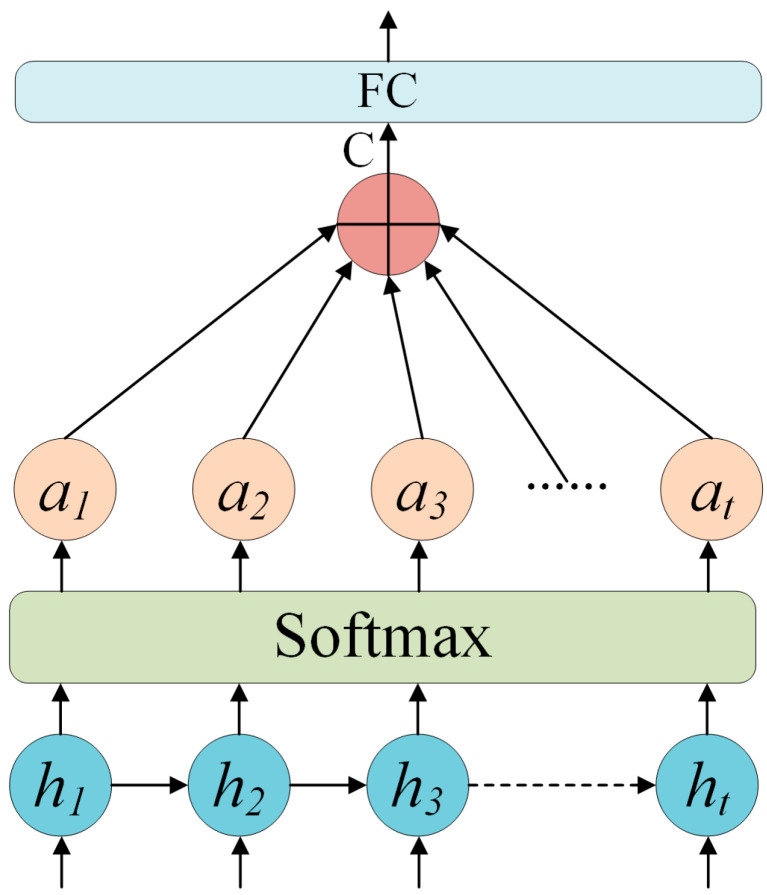
Structure of the attention mechanism.

In Equation (10), the weighted feature vector c represents the final attention-based representation for stress prediction. It encapsulates critical temporal information within the ultrasonic signal that the model has learned to emphasize. As shown in Figure 13, the attention weight *a* is applied to compute a weighted summation of the original sequence features *h*, thereby producing a feature vector *c* that is subsequently passed through a fully connected layer to yield the final stress regression prediction.

### 3.2. Model Training Configuration

Mean squared error (MSE) was employed to quantify the discrepancy between the predicted and true stress values. Model optimization was performed using the AdamW optimizer with an initial learning rate of 0.001 and a weight decay of 1 × 10^−5^ to mitigate overfitting and enhance generalization. All hyperparameters were optimized through a series of experiments to determine their optimal values, ensuring the best training performance across different model architectures.

The training process was limited to a maximum of 1000 epochs, with a batch size of 128, promoting faster convergence while reducing gradient variance and enhancing training stability. A learning rate scheduler was employed to dynamically adjust the learning rate during training, thereby improving optimization efficiency and overall model performance. All training and inference were conducted on a high-performance NVIDIA RTX 4090 GPU with 24 GB of memory. This configuration enabled efficient large-scale model training within a reasonable timeframe while maintaining experimental reproducibility and practical applicability.

## 4. Comparison and Analysis of Performance Among Different Models

A series of comparative experiments were conducted to assess the effectiveness of the proposed model and identify the optimal model for ultrasonic stress measurement. The evaluation included CNN of varying depths, alongside hybrid architectures combining recurrent neural networks and attention mechanisms. All models were trained on the same dataset under identical training parameters. An early stopping criterion based on validation performance was applied, automatically terminating training if no improvement was observed for 150 consecutive epochs. This approach effectively prevented overfitting while ensuring optimal performance for each model.

Model performance was assessed using three standard regression metrics: mean absolute error (MAE), root mean square error (RMSE), and the coefficient of determination (R^2^). MAE represents the average absolute difference between predicted and true values, reflecting overall prediction accuracy. RMSE measures the degree of fluctuation in prediction errors and is more sensitive to abnormal predictions. R^2^ is used to assess the model’s goodness of fit, with values closer to 1 indicating a stronger ability of the model to capture the relationship between ultrasonic signals and stress.

The performance of each model is summarized in Table 1. To systematically identify the contribution of each architectural component including network depth, receptive field size, temporal modeling strategies, and attention mechanisms, a step-by-step ablation analysis was conducted based on the progressive optimization of the model structure. Initially, the impact of the convolutional architecture was evaluated. The baseline 3-layer CNN achieved a mean absolute error of 10.34 MPa. Increasing the depth to a 5-layer CNN reduced the MAE to 8.24 MPa confirming that deeper networks are more effective at extracting abstract hierarchical features. Subsequently, expanding the convolutional kernel size in the 5-layer CNN with larger receptive field further decreased the MAE to 5.14 MPa. This significant improvement indicates that a wider receptive field is essential for capturing the global structural characteristics of the LCR waveform rather than relying solely on local peaks.

To capture the sequential evolution of ultrasonic signals, we further investigated different temporal modeling strategies. The CNN combined with BiLSTM and attention architecture achieved an MAE of 2.56 MPa. While effective, the bidirectional complexity increased computational cost without surpassing the simpler GRU. Similarly, the model integrating LSTM and the transformer achieved an MAE of 2.98 MPa likely because the self-attention mechanism of the transformer showed limited advantage in modeling the short-term dependencies of ultrasonic echoes given the dataset scale. Most notably, the integration of the Gated Recurrent Unit yielded the best performance with the 5-layer CNN and GRU model achieving an MAE of 1.42 MPa. This demonstrates that the GRU provides the optimal balance between capturing temporal dependencies and model simplicity for this specific task.

Finally, the proposed 5-layer CNN combined with GRU and attention model yielded an MAE of 1.94 MPa. Although this global error is slightly higher than the pure GRU model on the standard test set, the attention mechanism was explicitly retained to enhance the generalization capability of the model on unseen data. As detailed in the generalization tests in Section 5.2, the pure GRU model tends to overfit to the specific stress points in the training set. In contrast, the attention module effectively highlights intrinsic stress-sensitive regions, preventing performance degradation when predicting unseen stress states such as 35 MPa and 170 MPa, thereby ensuring the robustness of the model in practical applications.

In summary, the ablation analysis confirms that the hierarchical integration of deep convolutional features, GRU-based temporal modeling, and attention mechanisms is essential for accurate ultrasonic stress measurement. The progressive optimization of the network architecture not only improves the feature extraction capability but also ensures reliable generalization performance beyond the training data distribution.

## 5. Analysis of Model Visualization Results

### 5.1. Model Performance Analysis

To further assess the generalization ability and predictive stability of the proposed model under previously unseen stress conditions, a stress value removal experiment was conducted. Samples with stress values of 35 MPa, 80 MPa, 125 MPa, and 170 MPa were randomly removed from the training set and used exclusively for testing. The trained model’s performance was comprehensively assessed using scatter regression analysis, error distribution statistics, and residual cloud analysis.

**Figure 14:**
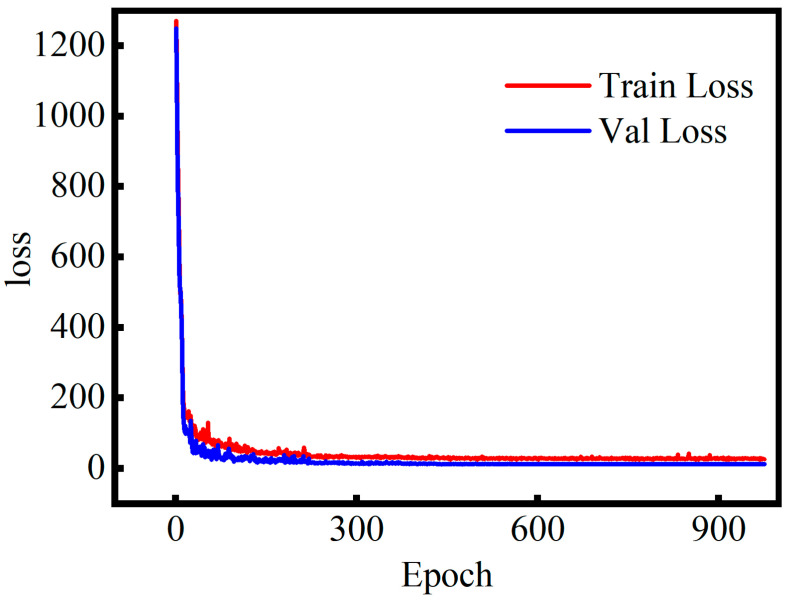
The current model’s training and validation loss curves.

Figure 14 shows the evolution of the loss function during training and validation. The loss decreases sharply in the initial epochs and converges rapidly at approximately 200 epochs. Thereafter, both curves gradually plateau and stabilize around 600 epochs. The close alignment of the training and validation loss trends, without significant oscillations or divergence, indicates the absence of overfitting. These results validate the effectiveness of the training strategy and confirm the soundness of the model optimization process.

**Figure 15:**
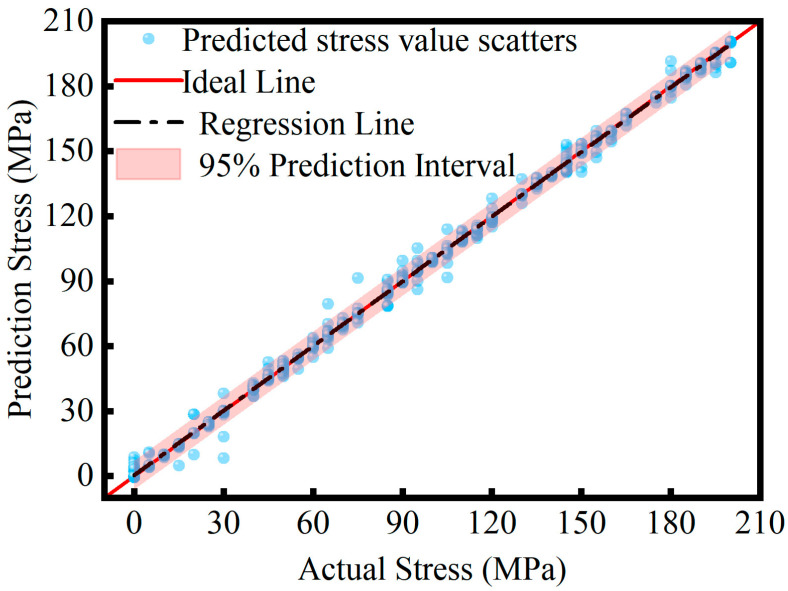
Actual stress values and predicted stress values.

Figure 15 shows the scatter regression plot of predicted versus actual stress values on the test set. The red ideal line denotes the theoretical perfect prediction where the predicted stress is exactly equal to the actual stress, y = x, while the black dashed line represents the regression fit of the predicted values. Blue data points cluster tightly along the ideal line, with the regression line nearly coinciding with it, demonstrating strong linearity across the 0–200 MPa stress range. No fitting imbalance is observed in either the low-stress or high-stress intervals, and no systematic offset or slope deviation is apparent. Further analysis shows that the 95% prediction interval, representing the confidence range of the model’s outputs, encompasses the vast majority of data points. The interval exhibits uniform width and smooth variation across the entire data range, without noticeable local fluctuations. These results indicate that the proposed model not only achieves high fitting accuracy but also maintains stable uncertainty control, ensuring precise and reliable stress predictions.

**Figure 16:**
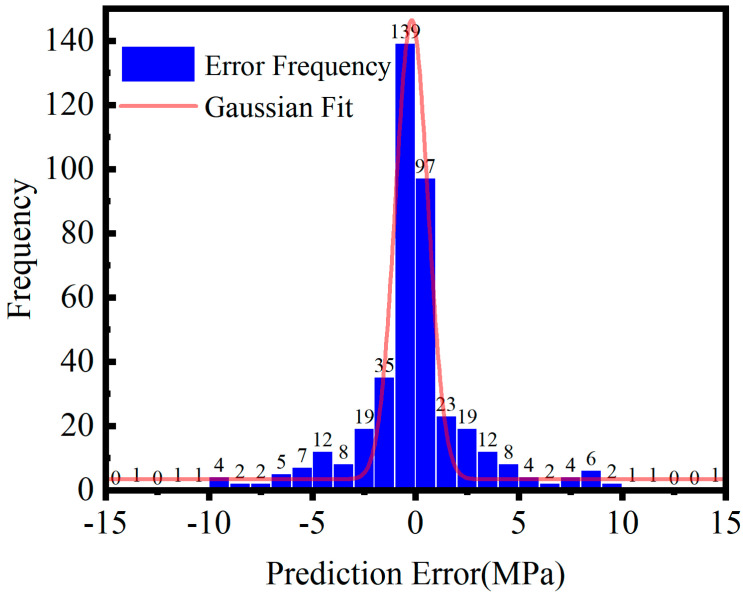
Histogram of prediction error distribution.

Figure 16 shows the histogram of prediction errors on the test set, illustrating the overall error distribution and concentration. The distribution closely follows a normal pattern, with its primary peak centered near zero, consistent with the ideal error pattern of a regression model. Notably, 90% of the data exhibit errors within ±5 MPa. Among 416 samples, 236 have absolute errors below 2.5 MPa, accounting for 56.7% of the total, indicating that most predictions are highly accurate with minimal deviations. Further analysis shows that the error distribution exhibits high kurtosis and near-zero skewness, suggesting a concentrated and symmetric pattern with a very low proportion of outliers. This implies that the model’s prediction errors primarily originate from inherent modeling uncertainty and environmental noise, rather than from systematic structural deficiencies or biases induced by overfitting. Additionally, the proportion of samples with errors exceeding ±10 MPa is minimal, confirming the model’s strong robustness in suppressing outliers and maintaining stable predictive accuracy.

**Figure 17:**
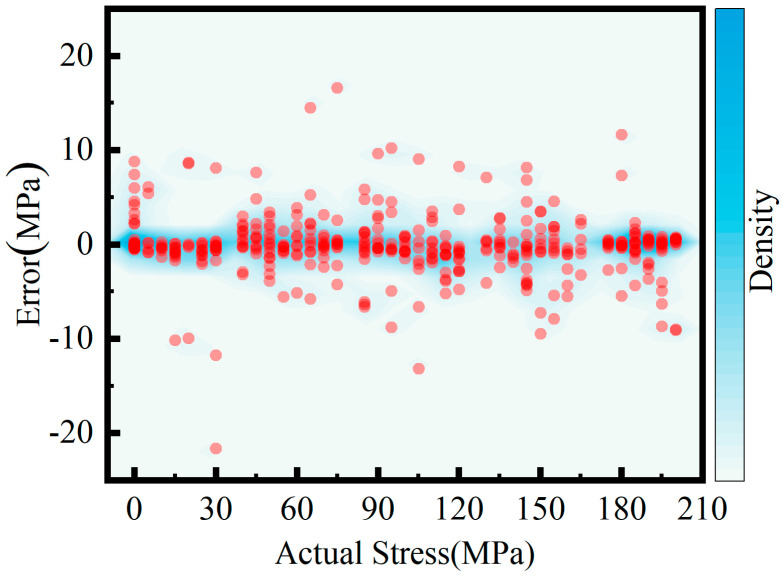
Scatter plot of prediction errors.

Figure 17 shows the two-dimensional distribution of the model’s predicted residuals with respect to actual stress levels. The residuals are predominantly concentrated within ±2.5 MPa and are uniformly distributed across the entire stress value range, showing no evident bias. The model maintains consistent prediction accuracy across low, medium, and high stress levels, with no error amplification at high stress or instability at low stress, confirming the model’s stability and reliability. The density distribution peaks near zero residual, indicating that most predicted values exhibit minimal deviations with well-controlled error fluctuations. A small number of residual points fall outside the ±5–10 MPa range. However, they are sparse, randomly scattered, and show no clustering or regional anomalies, exerting negligible influence on overall prediction stability.

Comprehensive analyses show that the 5-layer CNN+GRU+Attention model achieves excellent fitting ability, error control ability and predictive performance. Overall prediction accuracy satisfies the requirements of a high-precision regression model.

### 5.2. Generalization Ability Evaluation of the Model

This section systematically assesses the generalization ability of the proposed model by comparing the predictive performance of the 5-layer CNN+GRU model with and without the attention mechanism. The assessment focuses on four stress levels, 35 MPa, 80 MPa, 120 MPa, and 170 MPa, which were intentionally excluded from training sets. These data span the entire stress values range, enabling a comprehensive evaluation of the model’s generalization ability. For each stress level, 10 independent test samples formed a separate test set. This ensured that predictions were entirely independent of the training data. As shown in Table 2, the models incorporating attention mechanisms yield average predictions that more closely match the actual stress values. Notably, at the boundary of the 35 MPa and 170 MPa stress intervals, the prediction errors of stress values decreased markedly from 7.53 MPa and 7.72 MPa for models without attention to 3.18 MPa and 3.62 MPa, representing a reduction of over 50%. This improvement arises not from differences in training data volume but from the attention mechanism’s capacity to enhance feature extraction in boundary regions. Consequently, the model effectively captures stress-related information even where ultrasonic signal variations are subtle, thereby overcoming a common limitation of deep learning models in predicting at the boundary regions of the training range.

**Figure 18:**
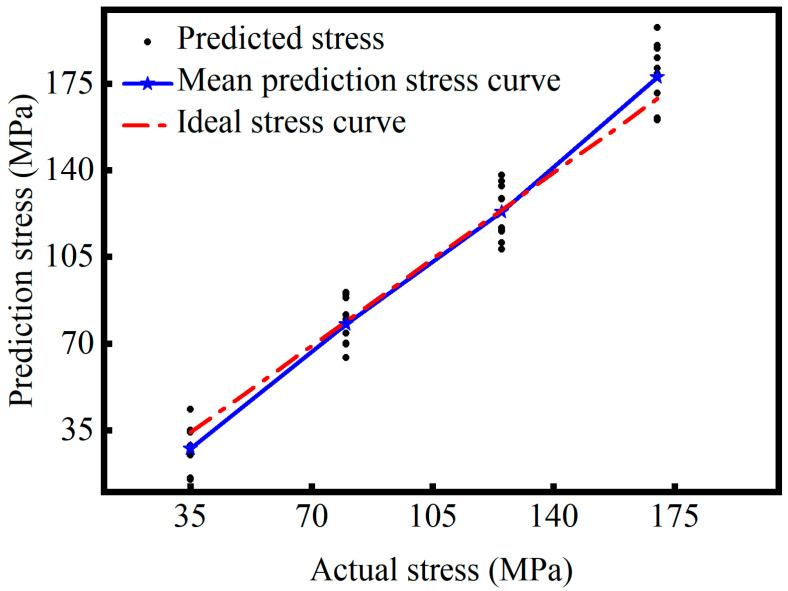
5-layer CNN+GRU model.

**Figure 19:**
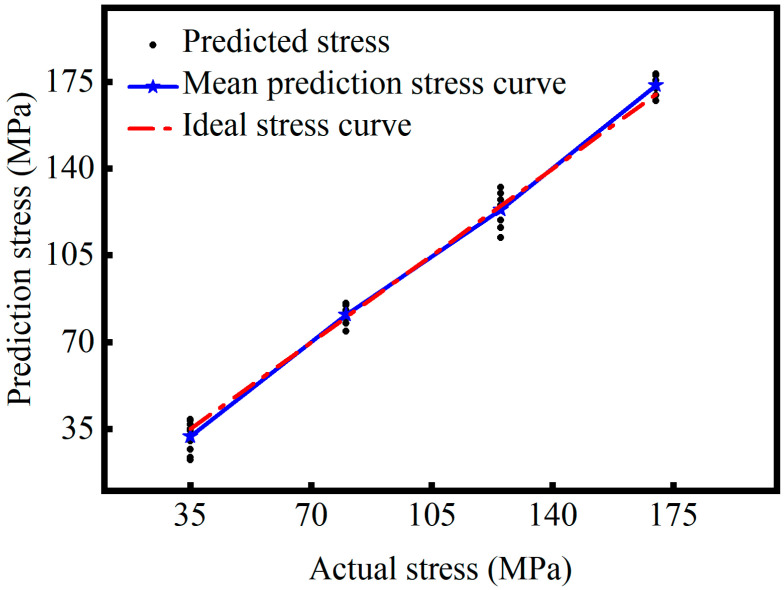
5-layer CNN+GRU+Attention model.

Further analysis of Figure 18 and Figure 19 provides a more intuitive understanding of the prediction performance of the two model configurations across different stress levels. As shown in Figure 18, the model without the attention mechanism produces relatively concentrated predictions with good linearity at mid-range stress levels. The red dashed line in the figure represents the ideal stress curve, which corresponds to y = x, indicating that the predicted values are equal to the true values. However, at 35 MPa and 170 MPa, the scatter points diverge, and several predictions deviate from the ideal stress curve, indicating unstable and highly fluctuating performance under boundary stress conditions. This may be because the amplitude of the ultrasonic signal changes little and is close to the noise level in the low stress conditions. At high stress levels, changes in material microstructure and acoustic propagation characteristics make it challenging for the model to effectively identify stress-dependent signal features. In contrast, as shown in Figure 19, the attention-based model exhibits densely clustered prediction points across all stress levels. Particularly at the boundary regions, the predicted values align closely with the ideal curve and remain uniformly distributed without significant deviations. This demonstrates that the attention mechanism enhances the model’s adaptability under low signal-to-noise ratio and non-ideal conditions. Furthermore, the fitted blue curve closely follows the ideal curve, confirming that the model achieves high prediction accuracy and strong generalization ability.

Comprehensive analysis reveals that the attention-based model maintains consistently high prediction accuracy across both the central and the low and high stress regions of the training range. The average error remains below 3 MPa, satisfying the dual requirements of precision and robustness for industrial nondestructive testing. Conversely, while the model without attention achieves a slightly lower mean absolute error of 1.42 MPa on the standard test set, it exhibits larger errors exceeding 7.5 MPa and poorer fitting at low and high stress levels, limiting its applicability under complex stress distributions. From an engineering standpoint, steel structures in service are often subjected to highly variable stress levels. Traditional models tend to fail under these conditions, particularly during early low-load or extreme high-load phases. Sacrificing a minor 0.52 MPa of average accuracy to prevent severe prediction failures at low and high stress levels is a necessary and beneficial strategic balance. Ultimately, the attention mechanism exhibits superior prediction stability, effectively fulfilling the strict requirements of reliable stress measurement under diverse operating conditions.

### 5.3. Comparative Analysis with Conventional Acoustoelastic Theory

To comprehensively evaluate the advantages of the proposed deep learning model, its predictive performance was compared against the traditional analytical approach. To provide a rigorous benchmark for the experimental results in Table 3, the conventional stress evaluation method is detailed here. This approach relies on the acoustoelastic effect where the absolute stress σ is calculated from the propagation time variation as follows

(11) $$\sigma =\frac{t-t_{0}}{K\times t_{0}}$$

In this expression, *t*_0_ denotes the propagation time in the reference stress free state while *t* represents the time under a specific load and *K* is the acoustoelastic coefficient calibrated for the 20# steel specimens. The time-of-flight variation is precisely determined using a cross-correlation algorithm to identify the temporal shift between the reference and target signals. The cross-correlation function *R_xy_*(*τ*) is defined by the following integral

(12) $$R_{xy}(\tau )=\int_{-\infty }^{+\infty }x(t)y(t+\tau )dt$$

The optimal time delay is obtained by finding the lag τ that maximizes the similarity between the two signals. This conventional method of calculating stress through cross-correlation based time-of-flight measurement has been widely applied in the research of Li et al. [12] and Liu et al. [17]. These established methods serve as a rigorous baseline for the comparative analysis presented in this study.

**Figure 20:**
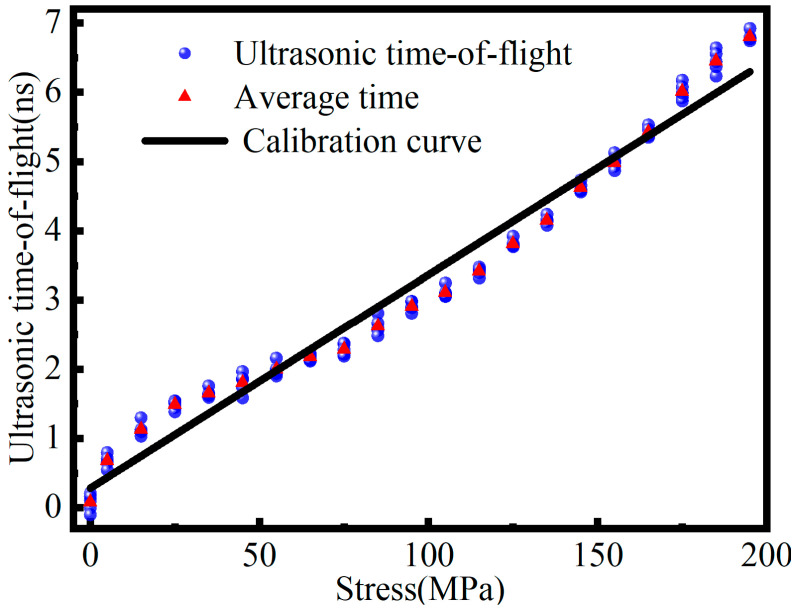
Linear regression of ultrasonic time-of-flight versus tensile stress.

While this analytical method provides a fundamental baseline, a critical limitation is evident in its practical application. To ensure a fair comparison, the conventional linear model was calibrated using the original unaugmented signals from the training dataset. As observed in Figure 20, at each stress increment within this training set, five independent ultrasonic time-of-flight measurements were recorded and are represented by the blue dots. The red triangles denote the average value calculated from these five trials, which serves as the basis for the linear regression fit shown as the black solid line, corresponding to an acoustoelastic coefficient *K* was 3.4498 × $10^{-6}$ MPa^−1^. The comparative predictions in Table 3 were then generated entirely from an independent test set.

Prior to the conventional calculations, every individual signal was hardware-averaged 128 times and digitally filtered to suppress noise. It is evident that the average values indicated by the red triangles deviate noticeably from the linear fitted line, particularly in the low- and high-stress regions, highlighting the limitations of a simple linear calibration. Furthermore, the individual raw measurements shown as blue dots exhibit distinct stochastic fluctuations around the average trend. Despite these processing steps, the conventional method still exhibited significant errors on the independent test set. This indicates that traditional cross correlation methods relying on a rigid calibration coefficient are highly sensitive to microscopic variations in transducer coupling states and environmental noise. The rigid linear formula relies on the averaged trend and inherently treats these raw fluctuations as errors to be discarded. However, in single-shot field measurements where averaging is not feasible, these deviations lead to significant prediction errors. In contrast, the proposed deep learning model treats these raw signal variations not as noise but as high-dimensional features, implicitly compensating for the instability that the linear model fails to accommodate.

To quantify this precision gap, Table 3 presents a detailed comparison of the stress measurement results between the two methods across the range of 15 to 195 MPa. The conventional method, constrained by the fixed acoustoelastic coefficient, exhibits significant instability at specific stress points where signal fluctuations occur. For instance, at 115 MPa, the conventional calculation deviates drastically with an error of −16.51 MPa, corresponding to a relative error of 14.36%. Similarly, in the high-stress region at 195 MPa, the conventional method produces a large positive error of 15.36 MPa, with a relative error of 7.88%. These substantial errors clearly demonstrate the insufficient accuracy of the rigid linear model for precise stress evaluation.

In sharp contrast, the proposed deep learning model maintains consistently high precision across the entire loading spectrum. At the same challenging stress levels of 115 MPa and 195 MPa, the model restricts the prediction errors to negligible values of −0.18 MPa and 1.67 MPa, respectively, corresponding to relative errors of only 0.16% and 0.86%. Overall, the conventional approach yields a mean absolute error of 9.88 MPa with an average relative error of 17.92%, whereas the proposed 1D-CNN-GRU-Attention model achieves a significantly lower MAE of 1.08 MPa and an average relative error of only 1.90%. This represents an error reduction of approximately 89%, while the relative error is reduced by approximately 89.4%.

The 1.08 MPa error of our proposed architecture also represents an approximate 89% improvement over the 10 MPa measurement error reported in the research of Li et al. [12]. Furthermore, it delivers a 78% enhancement in accuracy compared to the 4.94 MPa error achieved in the study of Liu et al. [17]. In summary, this innovative method achieves a substantial leap in accuracy within the field of ultrasonic stress measurement.

It is important to emphasize that the dataset used for model training and the prediction data presented in Table 3 were derived from independent loading experiments. This strict separation ensures that the evaluation reflects the model’s true generalization capability rather than memorization of specific experimental instances. Regarding the scope of application, the specific model parameters established in this study are valid for 20# steel under room temperature conditions using LCR waves. However, the proposed deep learning framework, which integrates 1D-CNN, GRU, and attention mechanisms, is a general deep learning framework for ultrasonic stress detection. The architecture is not restricted to a single material or wave mode. For applications involving different materials, such as aluminum alloys and composites, or alternative ultrasonic modes including Shear waves and Lamb waves, the core network structure remains applicable. To extend the method to these new domains, the framework simply requires retraining with a corresponding dataset characteristic of the target material and environmental conditions.

## 6. Conclusions

To address the heavy reliance of ultrasonic stress measurement on manual calibration, this study proposes a stress measurement method based on deep neural networks. Based on LCR waves, an end-to-end stress measurement model was developed to directly map raw time series ultrasonic data to stress values, eliminating the need for traditional calibration curves. This approach markedly enhances automation and intelligence in stress measurement while improving adaptability under practical engineering conditions. Data acquisition was performed using a piezoelectric ultrasonic experimental platform coupled with a hydraulic loading unit. Ultrasonic signals were collected from 420 standard 20# steel specimens across a stress range of 0–200 MPa. Through data augmentation, the dataset was expanded to 4200 samples and partitioned into training, validation, and test sets at a ratio of 8:1:1. After multiple training iterations, the proposed 5-layer CNN+GRU+Attention model achieved an average absolute error of 1.94 MPa. For previously unseen stress values, prediction errors remained within 3 MPa. Overall, the model demonstrates high measurement accuracy, along with strong generalization and robustness. It provides an efficient and reliable technical solution for calibration-free ultrasonic stress measurement, with significant practical value and broad applicability for the safety assessment and structural health monitoring of steel structures.

Despite the high accuracy and promising generalization of the proposed model for piezoelectric ultrasonic stress measurement, several limitations remain. The experimental dataset primarily comprises 20# steel specimens, leaving the model’s adaptability to other materials and complex structures untested. While the current study demonstrates the effectiveness of the proposed model for standardized 20# steel components, the influence of material anisotropy in other materials remains an important factor. Future work will extend the proposed framework to anisotropic materials such as aluminum alloys and titanium alloys, further enhancing its capability for industrial applications. Specifically, future research will focus on enhancing the engineering applicability of the model through transfer learning techniques. By utilizing the current model as a pre-trained backbone, we aim to achieve rapid adaptation to different steel grades and complex acoustic environments with minimal additional data collection. Furthermore, the inherent black box nature of deep learning limits its physical interpretability. Therefore, future research will primarily investigate what specific stress related physical features the network has extracted.

## Figures and Tables

**Table 1:** Performance Metrics of Each Model.

Model	MAE (MPa)	RMSE (MPa)	R^2^
3-Layer CNN	10.34	17.95	0.9093
5-Layer CNN	8.24	11.18	0.9675
5-layer CNN (Larger Receptive Field)	5.14	7.72	0.9856
CNN+BILSTM+Attention	2.56	4.35	0.9873
CNN+LSTM+Transformer	2.98	4.86	0.9891
5-layerCNN+GRU+Attention	1.94	3.31	0.9973
5-layerCNN+GRU	1.42	3.15	0.9976

**Table 2:** Average Prediction Results of Two Models Under Unseen Stress Conditions.

Actual Stress (MPa)	35	80	125	170
Mean Prediction (MPa) (No Attention)	27.47	77.83	123.09	177.72
Relative Error (MPa)	−7.53	−2.17	−1.91	7.72
Mean Prediction (MPa) (With Attention)	31.82	80.96	123.36	173.62
Relative Error (MPa)	−3.18	0.96	−1.64	3.62

**Table 3:** Prediction comparison between the deep learning model and the conventional acoustoelastic method.

Actual Stress (MPa)	DL Model Predicted Stress (MPa)	DL Model Error (MPa)	DL Model Relative Error (%)	Conventional Method Calculated Stress (MPa)	Conventional Method Error (MPa)	Conventional Method Relative Error (%)
15	14.44	−0.56	3.73	26.52	11.52	76.8
35	31.65	−3.35	9.57	47.91	12.91	36.89
55	55.43	0.43	0.78	52.53	−2.47	4.49
75	73.23	−1.77	2.36	62.75	−12.25	16.33
95	95.58	0.58	0.61	85.02	−9.98	10.51
115	114.82	−0.18	0.16	98.49	−16.51	14.36
135	135.06	0.06	0.04	125.09	−9.91	7.34
155	156.32	1.32	0.85	153.40	−1.60	1.03
175	175.88	0.08	0.05	181.26	6.26	3.58
195	196.67	1.67	0.86	210.36	15.36	7.88
Mean Error		1.08 MPa	1.90%		9.88 MPa	17.92%

## Data Availability

The original contributions presented in this study are included in the article. Further inquiries can be directed to the corresponding author.
